# Predicting Dietary Impact on Multiple Sclerosis‐Related Symptoms With the Gut Microbiome: A Pilot Study Using Unsupervised Machine Learning

**DOI:** 10.1002/brb3.71394

**Published:** 2026-04-20

**Authors:** Leeann Aguilar Meza, Rachel L Fitzjerrells, Farnoosh Shemirani, Tyler J Titcomb, Linda M Rubenstein, Patrick Ten Eyck, Linda G. Snetselaar, Shailesh K. Shahi, Terry L Wahls, Ashutosh K Mangalam

**Affiliations:** ^1^ Holden Comprehensive Cancer Center University of Iowa Iowa City Iowa USA; ^2^ Interdisciplinary Graduate Program in Informatics University of Iowa Iowa City Iowa USA; ^3^ College of Dentistry University of Iowa Iowa City Iowa USA; ^4^ Department of Internal Medicine, Carver College of Medicine University of Iowa Iowa City Iowa USA; ^5^ Department of Epidemiology University of Iowa Iowa City Iowa USA; ^6^ Institute for Clinical and Translational Science University of Iowa Iowa City Iowa USA; ^7^ Department of Neurology University of Iowa Iowa City Iowa USA; ^8^ Department of Pathology Carver College of Medicine University of Iowa Iowa City Iowa USA; ^9^ Iowa City VA Health Care System Iowa City Iowa USA

**Keywords:** low‐saturated fat diet, multiple sclerosis, modified paleolithic elimination diet, machine learning, microbiome

## Abstract

**Background:**

Multiple sclerosis (MS) is a neurodegenerative disease where dietary intervention has emerged as a potential adjunct treatment. Recently, the modified Paleolithic elimination (MPE) diet, also known as the Wahls diet, and the low‐saturated fat (LSF) diet, also known as the Swank diet, were linked to reduced fatigue and improved quality of life (QoL) in the WAVES study (NCT02914964). However, how diet impacts these outcomes remains unclear. As diet impacts gut microbiota, we investigated whether the baseline gut microbiota can predict response to diet in people with MS (pwMS).

**Methods:**

We performed fecal 16s rRNA sequencing to profile the microbiome changes associated with pwMS receiving the MPE (*n =* 11) and LSF diet (*n =* 12). Next, we utilized topic modeling, a machine learning technique, to determine whether baseline microbiome features predicted diet response in the combined MPE *+* LSF dietary cohort (*n =* 23).

**Results:**

Specific genera significantly differed over time on both diets. On the MPE diet, *Hungateiclostridiaceae, Ruminiclostridium*, and *Shuttleworthia* decreased, while *Coriobacteriaceae Collinsella* decreased on LSF. Predictive machine‐learning analysis associated a baseline microbiome enriched with *Akkermansia, Bacteroides*, and *Barnesiella* with fatigue response in the combined MPE *+* LSF cohort. For a non‐response in Mental QoL improvement in the combined MPE *+* LSF cohort, our analysis associated an enrichment of *Faecalibacterium* and *Alistipes* at the start of the diet.

**Discussion:**

Utilizing topic modeling, this pilot study identified baseline microbiota communities linked to improvements in fatigue and Mental QoL in pwMS on dietary intervention. These findings highlight the microbiota's role in dietary response and the potential for personalized nutrition. Given the small cohort and exploratory design, the results are hypothesis‐generating and require validation in larger mechanistic studies.

## Graphical abstract

Machine learning based topic modeling identified associations between baseline gut microbiota and dietary response in pwMS. Identified distinct microbiota linked to fatigue improvement and Mental QoL non‐response. Highlights the potential for microbiome‐informed personalized diets, though findings are preliminary.

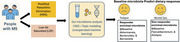

## 1 Introduction

Multiple sclerosis (MS), an inflammatory, demyelinating disease of the CNS, impacts 2.8 million people worldwide (Walton et al. 2020). The most common type of MS is relapsing‐remitting multiple sclerosis (RRMS), which is identified by periods of symptom flare‐up (relapse) and partial or full recovery (remission). In the past decade, disease‐modifying therapies (DMTs) for MS have vastly improved (Tintore et al. 2019). However, because many DMTs come with adverse side effects and are costly to the patients, many people with MS (pwMS) express interest in adjunct treatments, such as diet, to manage their symptoms (Dunn et al. 2015).

Several dietary approaches have been investigated as potential adjunct treatment options, both in animal models and in pwMS. One is the modified Paleolithic elimination (MPE) created by Dr. Terry Wahls, which excludes gluten‐containing grains, eggs, dairy, ultra‐processed food, and soy products and incorporates algae, nutritional yeast, and fermented foods. It emphasizes the consumption of foods that will support cellular health to reduce MS symptoms and improve quality of life (QoL) (Wahls et al. 2019; Evans et al. 2019; Lee et al. 2021; Irish et al. 2017). Another is the low‐saturated fat diet (LSF) developed by Dr. Roy Swank in 1948, who observed an association between consumption of saturated fat and higher incidence of MS (Swank 1970). Swank proposed that limiting saturated fats could improve cerebrovascular health and alleviate MS symptoms (Azary et al. 2018; Fitzgerald 2018). In fact, both diets do have preliminary evidence to support the alleviation of MS symptoms (Wahls et al. 2021). The prior study found significant improvements in fatigue and Physical and Mental QoL (MSQoL‐54) in pwMS on the MPE diet, with similar results for LSF. Mental QoL improved only in the MPE group (Wahls et al. 2021). However, the mechanisms underlying these effects remain unclear.

One plausible mechanism through which dietary intervention can provide health benefits involves the gut microbiota. It is well established that trillions of bacteria living in the human gut play an important role in maintaining health through regulation of digestion, metabolism, immune response, gut barrier integrity, and neurotransmitter (Mohajeri et al. 2018). Dysbiosis, an altered microbial composition and function, has been consistently reported in pwMS (Turner and Mangalam 2024). Since diet has the biggest impact on gut microbiota composition, the beneficial effects of diet might be mediated through the modulation of gut microbiota composition and function (Zhang 2022), or baseline microbiota can influence the response to diet. Several taxa, including *Akkermansia, Bacteroides, Prevotella*, and members of the Lachnospiraceae family, have been implicated in immune regulation, gut barrier integrity, and metabolic signaling, all of which may contribute to or protect from MS (Cuffaro et al. 2023; Nogal et al. 2021; Shaheen et al. 2025; Ordoñez‐Rodriguez et al. 2023). However, few studies have examined how diet‑driven microbiome changes relate to clinical outcomes in pwMS, and even fewer have attempted to predict dietary response using baseline microbiome profiles. In particular, gut bacteria influence key metabolite pathways such as short‑chain fatty acids (SCFAs), phytoestrogen metabolism, bile acid signaling, and tryptophan–indole pathways, all of which may contribute to the beneficial effects observed with dietary intervention.

To address this knowledge gap, we conducted a pilot microbiome study to determine how the two well‐known diets (MPE and LSF) influence the microbiota in pwMS and their symptoms. Using 16s rRNA sequencing, the gut microbiome was assessed at baseline, 12, and 24 weeks on each diet. Both ecological approaches and unsupervised machine learning techniques were utilized to determine microbial changes and outcome changes following the MPE or LSF diets. This integrative approach allowed us to examine not only individual microbial taxa but also co‑occurring microbial communities that may act collectively to influence host physiology. Because this is an exploratory analysis with a small sample size, our goal is to generate mechanistic hypotheses and position this work explicitly as hypothesis‑generating, identifying the potential of microbiome‑guided personalized dietary strategies in MS.

## 2 Methods

### 2.1 WAVES Human Subject Enrollment

**FIGURE 1:**
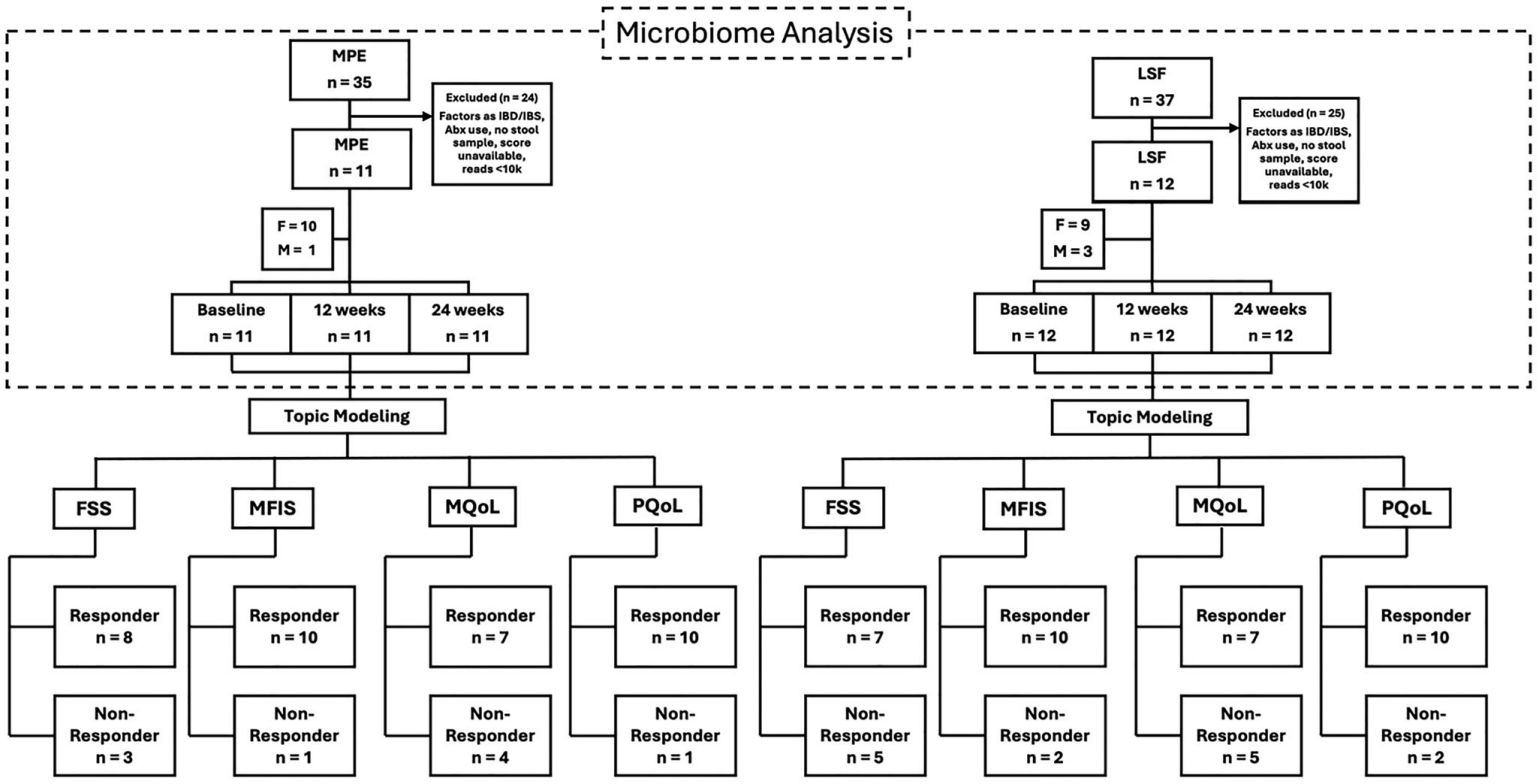
Diagram showing sample selection for analysis. All samples used for topic modeling were baseline samples, and their label (i.e., Responder or Non‐Responder) was based on their 24‐week response.

Participants were eligible for the parent trial based on published criteria (Wahls et al. 2021), including confirmed relapsing‐remitting MS (RRMS) (2010 McDonald), moderate to severe fatigue, residence within 500 miles of Iowa City, and dietary adherence. Exclusions included recent MS relapse, medication adjustments, low body mass index (BMI), pregnancy, severe mental impairment, and meeting other extensive criteria delineated in the study protocol (Wahls et al. 2018). As this pilot study investigated the microbiome, further exclusion criteria were applied, including antibiotic usage within three months of sample collection and gastrointestinal comorbidities (IBS/IBD) and diet non‐adherence (total *n =* 23). (Figure 1). Samples from previously consented parent‐study participants who met the microbiome exclusion criteria were included in the present microbiome pilot analysis (Table S1).

### 2.2 Diet Specifications

Participants received diet‐specific education from an RDN using self‐determination theory and motivational interviewing in individual and group sessions. Both groups followed their assigned diets ad libitum with identical supplements. The LSF diet restricted saturated fat to ≤15 g/day and included four servings of grains, fruits, and vegetables. The MPE diet included 6–9 servings of fruits and vegetables and 6–12 ounces of meat daily and excluded gluten, legumes, eggs, dairy (except ghee), soy, ultra‐processed foods, and nightshades, which were reintroduced after 12 weeks.

### 2.3 Human Fecal Sample Collection

Participants were instructed to provide fecal samples at baseline and follow‐ups at 12 and 24 weeks, with only those completing all visits included in this analysis. (Figure 1). At each visit, they were given an OMNI‐gene GUT stool collection kit (DNAgenotek Inc., Ottawa, Canada, Catalog #OMR‐200) to collect 1–3 days before the next study visit and bring to their next site visit. Samples were stored at −80°C.

### 2.4 DNA Extraction and Sequencing

16S rRNA sequencing of the V3‐V4 region was conducted on fecal samples following a published protocol (Shahi et al. 2019). DNA was extracted with the DNeasy PowerLyzer PowerSoil Kit (Qiagen) using bead‐beating. The V3‐V4 region was PCR‐amplified, barcoded with the Nextera XT Index Kit (Illumina), and sequenced on the Illumina MiSeq platform. The sequencing files were processed in R (version 4.2.2) (Team R. C. 2023) utilizing DADA2 (Callahan et al. 2016) with trimming of the forward and reverse reads at 280 and 240 bp, respectively. The Silva database (version 138.1) (Quast et al. 2013) was used for assigning taxonomy.

### 2.5 Microbiome Analysis

For microbiome analysis, phyloseq (v1.44.0) (McMurdie and Holmes 2013) was used. Samples with <10,000 reads were removed, taxa were filtered at >10 abundance in ≥25% of samples, and reads were CLR‐transformed. After processing, 95 Amplicon Sequence Variants (ASVs) remained. The average read count was 57,518 (MPE: min 23,089, max 88,638) and 55,498 (LSF: min 18,311, max 104,932). The dataset included 33 MPE samples (11 at each time point: baseline, 12 weeks, and 24 weeks) and 36 LSF samples (12 at each time point: baseline, 12 weeks, and 24 weeks), with analysis conducted at the genus level. Alpha diversity was assessed with the Shannon metric for microbial richness and evenness, while beta diversity was evaluated using Aitchison distance, with clustering significance tested via ANOSIM (vegan v2.6‐4) (Oksanen et al. 2022). Univariate analysis used a paired Wilcoxon test with a Benjamini‐Hochberg correction (p ≤ 0.05, FDR ≤ 0.25), and the Friedman test assessed population differences. Figures were generated with ggplot2 (v3.4.4) (Wickham 2016).

### 2.6 Topic Modeling the Gut Microbiome to Predict Diet Response

In Wahls 2021 (Wahls et al. 2021), the impact of LSF and MPE diets on fatigue and QoL was assessed using four metrics: Fatigue Severity Score (FSS), Modified Fatigue Impact Scale (MFIS), Physical and Mental Multiple Sclerosis Quality of Life Score 54 (QoL). Both diets significantly reduced FSS and MFIS scores, while Physical MSQoL improved on both, and Mental MSQoL improved only with MPE. The baseline microbiome as a predictor of diet response was investigated. The baseline samples, labeled by their 24‐week response to diet, “Responder” or “Non‐Responder,” were analyzed. A clinically meaningful response at 24 weeks was defined by the following criteria for each outcome:

**FSS**: decrease of at least 0.45 (Rooney et al. 2019).
**MFIS**: increase of at least 4 points (Rooney et al. 2019).
**Mental and Physical Quality of Life‐54**: Increase of at least 5 points for either (Jongen 2017).

Individuals without baseline samples were excluded (Figure 1). Each sample was assessed for four response outcomes: FSS, MFIS, Physical‐MSQoL, and Mental‐MSQoL. Responder status was defined using clinically meaningful change criteria from Wahls et al. (2021), including a ≥0.45 FSS decrease, ≥4‐point MFIS increase, and ≥5‐point improvement in either Mental or Physical MSQoL‐54. Topic modeling was performed to assess microbiome community types, as described previously (Fitzjerrells et al. 2024).

Because the aim was to capture overall co‐occurrence patterns rather than compare the diets directly, the MPE and LSF samples were combined to increase power. An initial exploratory analysis showed no significant differences in alpha and beta diversity between diets at any time point, suggesting similar microbiome composition over time (Figure 3A,B). Separate analyses were conducted for each patient‐reported outcome using the filtered phyloseq object. The ASV table was extracted, and the optimal topic number was determined with the FindTopicsNumber function from the LDAtuning package (version 1.0.2) (Nikita and Chaney 2020); with the minimization metrics CaoJuan2009 (Cao et al. 2009) and Arun2010 (Arun et al. 2010) (Figures 4A and 5A). Then using the Latent Dirichlet Allocation *(LDA)* function from the *topicmodels* package (version 0.2.14) (Grün and Hornik 2011), LDA was performed on the ASV table with *k* set to the ideal topic number identified in the previous step and *method = “VEM”* (Figures 4B and 5B). Using the *tidyverse* package (version 2.0.0) (Wickham et al. 2019), the beta (topic‐term assignment probability) and gamma (topic‐sample assignment probability) matrices were extracted. Adjacent to the term‐topic assignment figures are boxplots highlighting the relative abundance of assigned bacteria. This unsupervised machine‐learning approach identifies latent microbial co‐occurrence patterns (“topics”) undetectable by Wilcox tests, capturing features not significantly enriched or depleted in either group. Statistical analysis was performed in R (version 4.2.2). The topic modeling approach has been previously validated across several microbiome studies from our group (Fitzjerrells et al. 2024, Fitzjerrells et al. 2025, Ghimire et al. 2025), where it consistently identified stable, biologically meaningful microbial communities.

## 3 Data Availability

Raw metagenomic sequences can be found at the Sequence Read Archive (SRA) under BioProject ID PRJNA1083963. The metadata for the sequencing files is provided in Table S2.

## 4 Results

### 4.1 MPE and LSF Diets Induce Early Increases in Gut Microbiome Alpha Diversity, Followed by Stabilization

**FIGURE 2:**
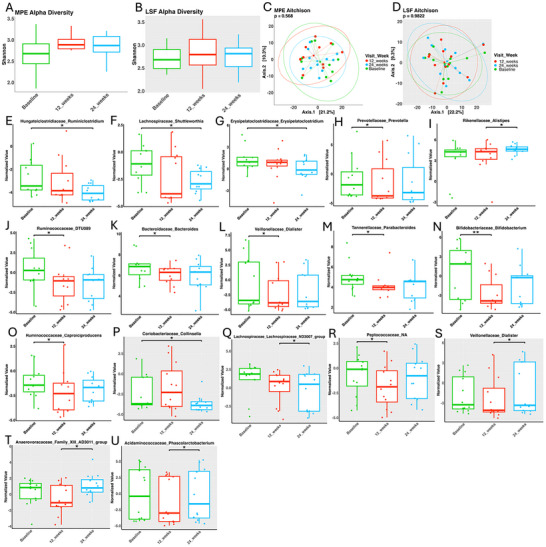
Microbiome diversity and individual bacterial changes on the MPE and LSF diets over time. Shannon diversity over time on the MPE (A) and LSF (B) diets. Aitchison dissimilarity over the course of the MPE (C) and LSF (D) diets. Eleven gut bacteria significantly change in abundance while on the MPE diet (E‐O). Six gut bacteria significantly change in abundance while on the LSF diet (P‐U). (* ≤ 0.05, ** ≤ 0.01, *** ≤ 0.001).

We assessed alpha diversity using the Shannon index to evaluate microbial richness and balance in response to the MPE and LSF diets over time. For the MPE group (Figure 2A), there were no significant changes in Shannon index values, though the median values showed an upward trend from baseline to 12 weeks. Similarly, the LSF group (Figure 2B) exhibited no major changes over time. However, the median Shannon index values slightly increased at 12 and 24 weeks, suggesting a gradual microbial adaptation to the diet.

**FIGURE 3:**
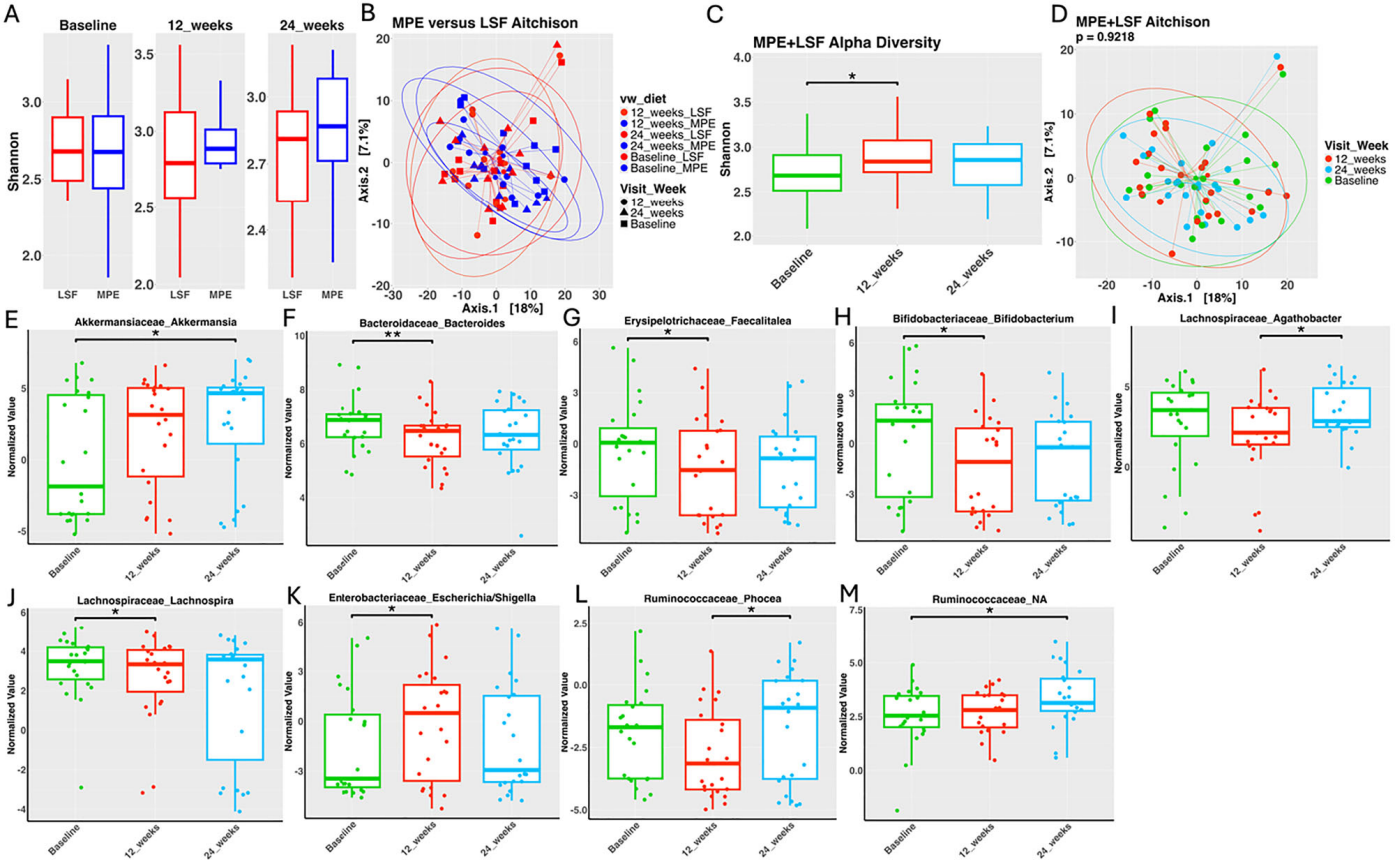
Comparison of MPE versus LSF at each time point and combined LSF + MPE diet groups. Alpha (A) and beta (B) diversity did not significantly differ between time points on these diets. Shannon diversity over time on diet (C). Aitchison dissimilarity over the course of diet (D). Nine gut bacteria significantly change in abundance while on a diet (E‐M). (* ≤ 0.05, ** ≤ 0.01, *** ≤ 0.001).

When comparing both groups (Figure 3C), Shannon index values significantly increased at 12 weeks compared to baseline (*p =* 0.021), indicating a similar early effect of both diets on microbial diversity. By 24 weeks, alpha diversity stabilized. These findings suggest that the diets initially (12 weeks) enhanced microbial richness and balance but seemed to settle into stable microbial communities by 24 weeks.

### 4.2 MPE and LSF Diets Induce Subtle Shifts in Gut Microbiota Composition and Diversity

Beta diversity, representing differences in microbial composition between groups, was assessed using Principal Component Analysis (PCA) based on Aitchison distances. In the MPE cohort (Figure 2C), no significant differences were observed (*p =* 0.568). However, the subtle shifts in microbial composition, particularly between baseline and 24 weeks, suggested the potential impact of dietary intervention on certain taxa. The LSF cohort (Figure 2D) and the combined cohorts (Figure 3D) show no significant differences in microbial composition.

### 4.3 Univariate Analysis Reveals Temporal Shifts and Microbial Community Structure in Response to Dietary Interventions

Taxonomic analysis revealed changes in microbial taxa across time points in both dietary groups and their combined analysis. In the MPE cohort (Figures 2E–O), several bacteria, including *Dialister* (*p =* 0.032), *Ruminococcaceae_DTUG09* (*p =* 0.014), *Prevotella* (*p =* 0.042), and *Bacteroides* (*p =* 0.042), showed significant reductions at 12 weeks but stabilized by 24 weeks. A second group of bacteria, such as *Caproiciproducens* (*p =* 0.019), *Bifidobacterium* (*p =* 0.010), and *Parabacteroides* (*p =* 0.024), also decreased significantly at 12 weeks but exhibited non‐significant upward trends by 24 weeks. Conversely, *Ruminiclostridium* (*p =* 0.042), *Shuttleworthia* (*p =* 0.032), and Erysipelatoclostridium (*p =* 0.024) demonstrated gradual but significant reductions, becoming apparent by 24 weeks. In contrast, *Alistipes* showed a gradual, non‐significant increase from baseline to 12 weeks, followed by a significant rise between 12 and 24 weeks (*p =* 0.042).

In the LSF cohort (Figures 2P–U), *Collinsella* (*p =* 0.043) and *Lachnospiraceae_ND3007_group* (*p =* 0.027) showed no significant changes from baseline to 12 weeks but decreased significantly by 24 weeks. *Peptococcaceae* exhibited a significant reduction from baseline to 12 weeks (*p =* 0.027) and a slight, non‐significant upward trend by 24 weeks. Meanwhile, *Dialister* (*p =* 0.043), *Family_XI_AD3011_group* (*p =* 0.027), and *Phascolarctobacterium* (*p =* 0.027) showed no significant changes between baseline and 12 weeks but increased significantly from 12 to 24 weeks.

Combined group analysis (Figures 3E–M) highlighted consistent patterns, with *Bacteroides* (*p =* 0.006), *Faecalitalea* (*p =* 0.048), *Bifidobacterium* (*p =* 0.014), and *Lachnospira* (*p =* 0.033) significantly decreasing at 12 weeks and stabilizing by 24 weeks (*p >* 0.05). *Agathobacter* levels trended downward overall but significantly increased between 12 and 24 weeks (*p =* 0.033). *Escherichia/Shigella* displayed a distinct pattern, significantly increasing from baseline to 12 weeks (*p =* 0.035) and trending downward by 24 weeks (*p >* 0.05). *Akkermansia* showed no significant changes from baseline to 12 weeks (*p >* 0.05) but significantly increased by 24 weeks (*p =* 0.035). *Ruminococcaceae_NA* displayed a steady upward trend, becoming significantly higher by 24 weeks (*p =* 0.048). These findings suggest that while the dietary interventions led to notable microbial adjustments, the patterns of change varied across taxa, with some stabilizing early and others showing delayed responses.

### 4.4 Topic Modeling‐Based Community Analysis Reveals Baseline Gut Microbiome as a Predictor of Patient‐Reported Outcome Responses to Diet

To enhance the statistical power for topic modeling analysis, the MPE and LSF diet groups were combined. Two patient‐reported outcomes showed significant associations with microbial community types: the FSS was linked to the responder topic, while Mental QoL was linked to a non‐responder topic. No significant associations were observed for Physical QoL or MFIS. A four‐topic structure was selected as the optimal model based on coherence metrics and statistical stability, ensuring the topics effectively captured meaningful microbial patterns across samples.

**FIGURE 4:**
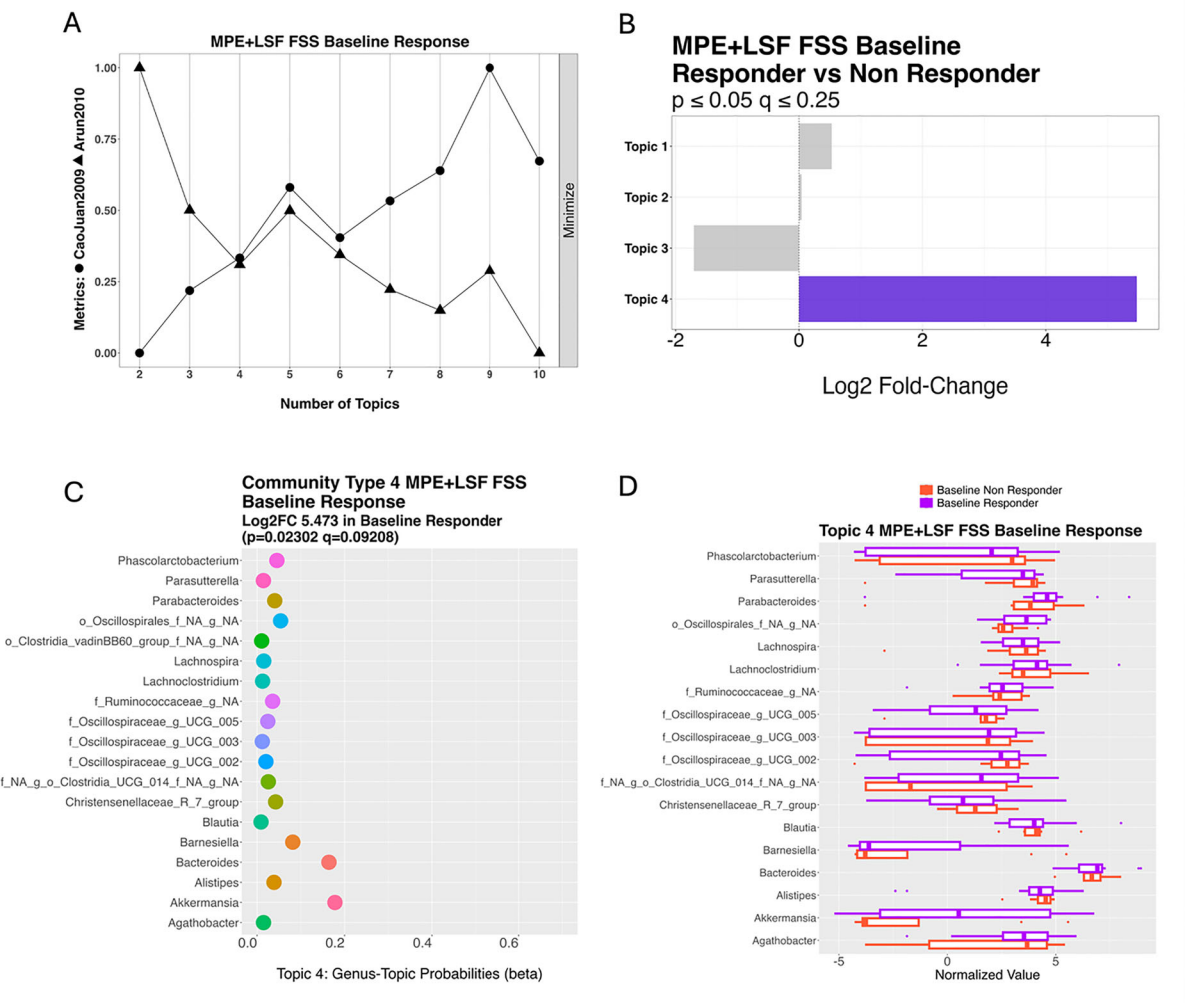
Microbiome community associated with FSS response on diet. Topic modeling of the microbiome community of the MPE + LSF Baseline samples future FSS response. (A) Assessment of ideal topic number. (B) Identification of significant topics (highlighted in purple). (C) Community significantly more often assigned to MPE + LSF FSS Responder group; baseline samples of individuals that did improve in FSS score at 24‐weeks. (D) Corresponding boxplot indicating relative abundance of microbes in topic.

For the FSS, Topic 4 was significantly associated with responders at baseline (*p =* 0.02, *q* = 0.09) (Figure 4B). Taxonomic analysis revealed that Topic 4 included a consortium of key taxa frequently co‐occurring across samples (Figure 4C). Prominent contributors included *Phascolarctobacterium*, *Parasutterella*, *Parabacteroides*, *Barnesiella*, *Akkermansia*, and *Alistipes*, along with members of the *Oscillospiraceae*, *Ruminococcaceae*, and families. Additionally, *Akkermansia* and *Bacteroides* were more abundant in responders. Boxplots of normalized values (Figure 4D) showed higher levels of these taxa in responders, with *Parabacteroides*, *Lachnoclostridium*, and *Barnesiella* being most consistently enriched. Elevated levels of *Akkermansia* and *Bacteroides* further characterized this responder‐associated community profile.

**FIGURE 5:**
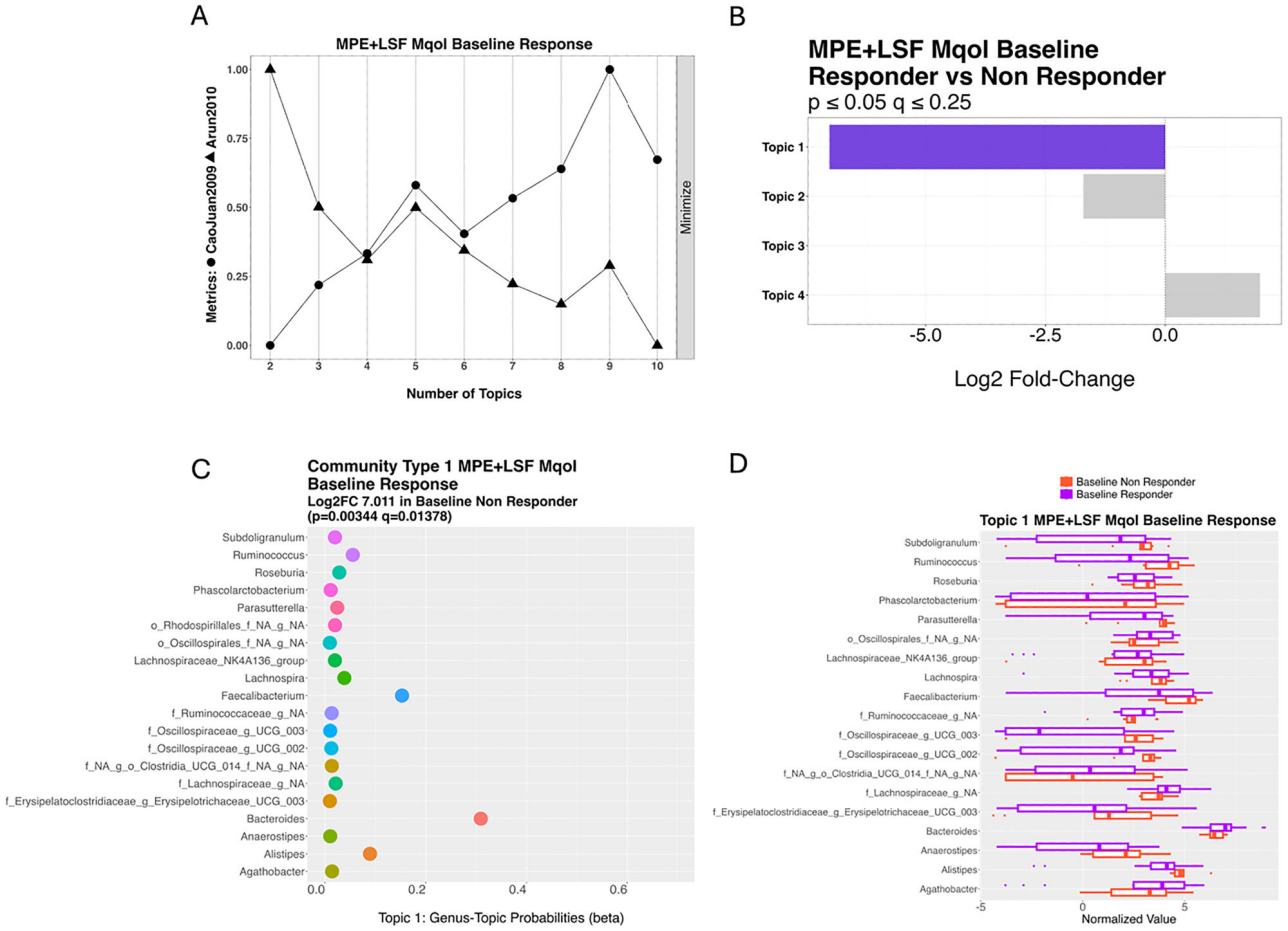
Microbiome community associated with Mental MSQoL non‐response on diet. Topic modeling of the microbiome community of the MPE + LSF Baseline samples future Mental MSQoL response. (A) Assessment of ideal topic number. (B) Identification of significant topics (highlighted in purple). (C) Community significantly more often assigned to MPE + LSF Mental MSQoL Non‐Responder group; baseline samples of individuals that did not improve in Mental MSQoL score at 24‐weeks. (D) Corresponding boxplot indicating relative abundance of microbes in topic.

In contrast, for Mental QoL, Topic 1 was significantly associated with non‐responders at baseline (*p =* 0.003, *q* = 0.01) (Figure 5B), comprised of taxa that commonly co‐occurred within this group (Figure 5C). Key contributors included *Ruminococcus*, *Subdoligranulum*, *Roseburia*, *Erysipelatoclostridiaceae_UCG_003*, *Parasutterella*, and *Anaerostipes*, along with members of the *Ruminococcaceae* families. Additional prominent taxa included *Bacteroides*, *Alistipes*, and *Faecalibacterium*. Boxplots of normalized values (Figure 5D) demonstrated higher levels of these taxa in non‐responders, with *Ruminococcus* and *Subdoligranulum* being particularly elevated, followed by *Roseburia* and *Faecalibacterium*.

These findings suggest that the baseline microbiome composition may influence an individual's response to adjunct diet therapy. Assessing a patient's microbiome prior to initiating such therapy could help predict their likelihood of achieving a positive outcome, potentially saving time and resources while personalizing treatment strategies.

## 5 Discussion

Dietary interventions are gaining attention as potential adjunct treatments for managing MS symptoms, yet the underlying mechanisms remain incompletely understood. Our findings suggest that the MPE and LSF diets, previously linked to fatigue reduction and improved QoL in RRMS (Wahls et al. 2021), may exert their beneficial effects through gut microbiota modulation. Importantly, baseline microbiota composition may provide early signals on differential dietary responsiveness, highlighting the potential of microbiota‐informed personalization.

Diet significantly influences gut microbiota composition and function throughout life (Singh et al. 2017). From early colonization by Actinobacteria (e.g., *Bifidobacterium* spp.) and Firmicutes (e.g., *Lactobacillus* spp.) (Stewart et al. 2018) to the stabilization of Bacteroidetes and Firmicutes phyla dominance in adulthood, dietary changes continuously shape the microbiome (Stewart et al. 2018). This responsiveness to diet underscores the potential of targeted dietary interventions, such as the MPE and LSF diets, for MS symptom management. Although alpha and beta diversity did not significantly shift over time, the modest early changes suggest that longer adherence or additional functional resolution may be needed to detect larger ecological effects. Univariate analyses revealed decreases in *Ruminoclostridium*, *Shuttleworthia*, and *Erysipelatoclostridium* on MPE, taxa enriched in pwMS, and reductions in *Collinsella* on LSF, consistent with prior MS literature.

With the ecological assessment of each diet, alpha and beta diversity did not significantly vary over time on either diet. However, as diet influences the microbiome, these observations suggest that a longer adherence to the dietary intervention might be necessary to observe significant changes in these ecological metrics.

Individuals on the MPE diet had significant changes in several bacteria. Looking at taxa that changed from baseline to 24 weeks, *Hungateiclostridiaceae Ruminoclostidium, Lachnospiraceae Shuttleworthia*, *Erysipelatoclostridiaceae, and Erysipelatoclostridium* decreased in abundance after being on the MPE diet. *Erysipelatoclostridium* has previously been identified as increased in patients with MS compared to healthy controls (Thirion et al. 2023). A decrease over time on MPE could be one way MPE imparts its beneficial effects on MS‐related symptoms. Additionally, MS patients on the Mediterranean diet and a brief high‐impact multidimensional rehabilitation program saw MS symptom improvement and improved quality of life. These changes were also associated with reduced *Ruminococcus* genera (Bronzini et al. 2023). On the LSF diet, *Coriobacteriaceae Collinsella* significantly decreased over time. Prior MS studies have identified a lower abundance of *Collinsella* in MS patients compared to healthy controls (Jangi et al. 2016; Chen et al. 2016).

To capture community‐level microbial patterns not evident in univariate analyses, we applied topic modeling, which identifies co‐occurring taxa that may act as functional units. Two baseline microbial communities were associated with differential clinical response across both diets: one linked to FSS improvement and another linked to non‐response in Mental QoL. The FSS responder community was enriched in *Akkermansia*, *Bacteroides*, *Barnesiella*, and *Alistipes*. Interestingly, *Akkermansia* species have been reported in both pathogenic and beneficial contexts in MS–some studies show increased abundance in pwMS compared with controls, whereas others link higher *Akkermansia* levels to reduced disability in RRMS and progressive MS (Cox et al. 2021). *Akkermansia* species, particularly *A. muciniphila*, has been shown to strengthen gut barrier integrity, promote mucus‐layer renewal, modulate host metabolic pathways, and induce regulatory immune responses through SCFA production, mechanisms that may contribute to improved fatigue outcomes in responders (Shaheen et al. 2025). However, when mucin degradation exceeds mucin production, excessive *Akkermansia* activity may thin the mucus layer and potentially promote inflammation through increased gut permeability, illustrating its context‐dependent effects (Ghimire et al. 2025; Peipert et al. 2025). *Bacteroides* and *Alistipe*s both produce SCFAs, which support intestinal epithelial cell growth, stabilize the gut barrier, and support host energy metabolism (Cuffaro et al. 2023; Parker et al. 2020; Wang et al. 2019; Mane et al. 2023; Lopez‐Siles et al. 2017) (Nogal et al. 2021). Their combined SCFA‐producing capacity may help explain fatigue improvements in responders. Thus, our pilot work suggests that individuals with higher baseline levels of *Akkermansia, Bacteroides, Barnesiella*, and *Alistipes* may be more likely to experience improvement in FSS scores due to the ability of these microbiota to enhance SCFA production, although larger studies are required to validate this association.

The Mental QoL non‐responder community was highly assigned *Faecalibacterium, Bacteroides*, and *Alistipes*. *Alistipes* was higher at baseline in the Mental QoL non‐responders, and although capable of producing beneficial SCFAs, it is also an indole producer; excess indole has been linked to reduced serotonin availability and detrimental gut–brain axis effects, including increased depressive symptoms (Parker et al. 2020). Consistent with this mechanism, a study of Norwegian patients with chronic fatigue syndrome found that increased *Alistipes* strongly correlated with depression, supporting the possibility that indole‐mediated effects on serotonin pathways may contribute to the lack of Mental QoL improvement in this group (Parker et al. 2020). *Bacteroides*, a major producer of acetate and propionate, was lower at baseline in non‐responders, suggesting reduced SCFA availability and potentially impaired barrier integrity. Lower SCFA levels have been linked with decreased serotonin production, which can disrupt gut–brain‐axis signaling and contribute to poorer Mental QoL outcomes. Although *Faecalibacterium* is a dominant butyrate producer and generally considered beneficial (Thirion et al. 2023), its higher abundance in non‐responders was somewhat puzzling, and the rationale for this association remains unclear. One possibility is that, as a dominant species, *Faecalibacterium* may stabilize or rigidify the microbial community, reducing its capacity to adapt to newly available dietary substrates. Taken together, the combination of higher *Faecalibacterium* and *Alistipes* with lower *Bacteroides* may reflect a baseline microbial community that is less responsive to dietary modulation, potentially contributing to the limited Mental QoL improvement observed despite dietary intervention. Because we used 16S rRNA sequencing, species‐ and strain‐level resolution was not possible, limiting our ability to attribute functional roles to specific microbial lineages.

These exploratory findings may suggest that MS symptom improvement could be linked to bacterial presence and proportion at the start of adjunct diet therapy. Identifying baseline microbiome patterns associated with favorable clinical response to specific diet manipulation could facilitate greater personalization of the diet recommendations for patients, pending future validation. In this way, pwMS that would have a higher likelihood of a positive response to either the MPE or LSF diet could be identified. In the future, it may be possible for clinicians to assess baseline gut microbiome characteristics and which diet pattern the person is most likely to benefit from.

Taken together, these community‐level associations suggest that dietary responsiveness in MS may depend on the functional capacity of the baseline gut microbiota. Taxa involved in SCFA production (e.g., *Bacteroides, Alistipes*, and *Faecalibacterium*) and mucin‐degradation or barrier‐support functions (*Akkermansia*) may create a metabolic environment more capable of utilizing substrates provided by the MPE or LSF diets, thereby enhancing fatigue‐related benefits. In contrast, communities enriched with indole‐producing species may negatively influence gut–brain axis signaling and blunt improvements in Mental QoL. These mechanistic patterns remain hypothesis‐generating and must be interpreted cautiously, as our small sample size and exploratory topic‐modeling approach were not powered to identify predictive microbial signatures. Nonetheless, this pilot study provides foundational insight into how baseline microbiota composition may shape clinical responses to dietary interventions in pwMS and highlights the potential for microbiota‐informed personalization. Future studies with larger and more diverse cohorts, longer dietary exposure, and species‐resolved metagenomics and metabolomics will be essential to validate these associations and define how diet‐related microbial changes contribute to fatigue, immune function, and broader MS symptom trajectories.

## Author Contributions

**Leeann Aguilar Meza**: investigation; writing – original draft; visualization; formal analysis; writing – review and editing. **Rachel L. Fitzjerrells**: formal analysis; writing – original draft; visualization; writing – review and editing. **Farnoosh Shemirani**: writing – review and editing; data curation. **Tyler J. Titcomb**: writing – review and editing; data curation. **Linda M. Rubenstein**: writing – review and editing; data curation. **Patrick Ten Eyck**: writing – review and editing; data curation. **Linda G. Snetselaar**: writing – review and editing; data curation. **Shailesh K. Shahi**: writing – review and editing; resources; methodology. **Terry L. Wahls**: writing – review and editing; funding acquisition; conceptualization; project administration; supervision; resources. **Ashutosh K. Mangalam**: writing – review and editing; funding acquisition; conceptualization; project administration; resources; supervision.

## Funding

The authors acknowledge funding from the NIH/NIAID 1RO1AI137075 (A.K.M.), Veteran Affairs Merit Award 1I01CX002212 (A.K.M.), University of Iowa Environmental Health Sciences Research Center, NIEHS/NIH P30 ES005605 (A.K.M.), a gift from P. Heppelmann and M. Wacek (A.K.M.), the Carver Trust Pilot Grant (A.K.M.), and a pilot award from the Center for Biocatalysis and Bioprocessing (CBB) (A.K.M.). R.L.F. was funded by the NIH 1F31DE033564‐01. This study was also supported in part by the National Multiple Sclerosis Society grant RG‐1506‐04312 (T.L.W.); the National Center for Advancing Translational Sciences of the NIH under Award Number UM1TR004403; and University of Iowa institutional funds. T.J.T. and F.S. are supported by the Carter Chapman Shreve Family Foundation and the Carter Chapman Shreve Fellowship Fund for diet and lifestyle research conducted by the Wahls Research team at the University of Iowa. T.J.T. was also supported by the Helen Harris Fund. In‐kind support was provided by the University of Iowa College of Public Health Preventive Intervention Center.

## Conflicts of Interest

A.K.M. is one of the inventors of a technology claiming the use of *Prevotella histicola* to treat autoimmune diseases. A.K.M. received royalties from Mayo Clinic (paid by Evelo Biosciences). However, no fund or product from the patent was used in the present study. T.L.W. personally follows and promotes the Wahls diet. She has equity interest in the following companies: Terry Wahls LLC, TZ Press LLC, The Wahls Institute PLC, FBB Biomed Inc., Foogal Inc., Levels Health Inc., and the website http://www.terrywahls.com. She also owns the copyright to the books Minding My Mitochondria (second edition) and The Wahls Protocol, The Wahls Protocol Cooking for Life, and the trademarks. The Wahls Protocol and Wahls diet, Wahls Paleo diet, and Wahls Paleo Plus diets (the Wahls elimination diet is not trademarked); and Wahls Behavior Change. She has completed grant funding from the National Multiple Sclerosis Society for the Dietary Approaches to Treating Multiple Sclerosis‐Related Fatigue Study. She has financial relationships with Standard Processes Inc., Vibrant America LLC, Great Plains Laboratory LLC, Mosaic Diagnostics LLC, and the Institute for Functional Medicine. Well Theory Technologies Inc, Levels Health Inc, FBB Biomed Inc and Foogal Inc. She receives royalty payments from Penguin Random House. T.L.W. has conflict of interest management plans in place with the University of Iowa and the Iowa City VA Health Care System. All other authors report no personal or financial conflicts of interest in this work.

## Supporting information




**Supplementary Table**: brb371394‐sup‐0001‐tableS1.xlsx

**Supplementary Table**: brb371394‐sup‐0001‐tableS2.xlsx

## Data Availability

The data that support the findings of this study are openly available in NCBI BioProject database at https://dataview.ncbi.nlm.nih.gov/object/PRJNA1083963?reviewer = vog4rq2tvhg625j2pq1aiolct5, reference number BioProject ID PRJNA1083963.
